# Incidence of malignant hyperthermia in patients undergoing general anesthesia: Protocol for a systematic review and meta-analysis

**DOI:** 10.1097/MD.0000000000009115

**Published:** 2017-12-08

**Authors:** Junyong In, Eun Jin Ahn, Dong Kyu Lee, Hyun Kang

**Affiliations:** a Department of Anesthesiology and Pain Medicine, Dongguk University Ilsan Hospital, Gyeonggido; b Department of Anesthesiology and Pain Medicine, Inje University Seoul Paik Hospital; c Department of Anesthesiology and Pain Medicine, Guro Hospital, Korea University School of Medicine; d Department of Anesthesiology and Pain Medicine, Chung-Ang University College of Medicine, Seoul, Republic of Korea.

**Keywords:** anesthesia, general, incidence, malignant hyperthermia, meta-analysis, protocol and guidelines, systematic review

## Abstract

**Background:**

Malignant hyperthermia (MH) continues to be of potential concern for clinicians whenever inhalational anesthetic agents or succinylcholine are used, because MH is a potentially fatal metabolic disorder.

**Methods:**

A systematic and comprehensive search will be performed using MEDLINE, EMBASE, and Google Scholar, for studies published up to November 2017. Peer-reviewed prospective cohort studies, retrospective cohort studies, and cross-sectional studies or reports issued by government organizations reporting the incidence or prevalence of MH will be eligible for inclusion. The quality of included studies will be assessed using the Newcastle–Ottawa scale and the modified risk of bias tool. Heterogeneity of estimates across studies as well as publication bias will be assessed. This systematic review and meta-analysis will be performed according to the Meta-analysis of Observational Studies in Epidemiology (MOOSE) guidelines and reported according to the Preferred Reporting Items for Systematic reviews and Meta-Analysis (PRISMA) guidelines. All statistical analyses will be conducted using the Stata SE version 15.0.

**Results:**

The results of this systematic review and meta-analysis will be published in a peer-reviewed journal.

**Conclusion:**

To our knowledge, this systematic review and meta-analysis will be the first to evaluate existing research on the incidence of MH. Our study will provide an overall estimate of the incidence of MH. Subgroup analysis will assess the incidence of MH according to age, gender, geographical region, race, and the provoking agent if possible. The review will benefit patients, healthcare providers, and policymakers.

**Ethics and dissemination:**

Ethical approval and informed consent are not required, as the study will be a literature review and will not involve direct contact with patients or alterations to patient care.

**Trial registration number:**

The protocol for this review has been registered in the PROSPERO network (registration number: CRD42017076628)

## Abstract 2

Supplemental Digital Content is available in the text

**Strengths and limitations**

- This systematic review and meta-analysis will provide a comprehensive and objective assessment of the incidence of malignant hyperthermia in patients undergoing general anesthesia and/or surgery.
- The study will provide useful and novel information for patients, healthcare providers, and policymakers.
- The study will assess the methodological and reporting qualities of included studies using the Newcastle–Ottawa scale and modified risk of bias tool.
- Our results may be limited by heterogeneity due to differences in age, gender, geographic region, race, and provoking agent.
- Subgroup analysis will be carried out based on quality of study, geographic region, age, gender, race, and provoking agent if possible.

## 1 Introduction

Malignant hyperthermia (MH) is a rare, yet potentially fatal disorder triggered by exposure to inhalational anesthetics (e.g., halothane, isoflurane, sevoflurane, desflurane, etc.) and succinylcholine. Even though mortality and morbidity have decreased over the past several decades, MH will continue to be of potential concern to clinicians whenever inhalational anesthetic agents or succinylcholine is used.

MH is a genetic disorder of skeletal muscle calcium regulation in humans, linked to the ryanodine receptor type 1 (RYR1) gene. Many studies from the past report an incidence of MH ranging from 1:10,000 to 1:220,000.^[1,2]^ This suggests that the precise incidence of MH is difficult to estimate due to its rarity and limited data. In addition, the incidence of MH seems to vary, depending on the geographic region, age, gender, and race.^[1,3–6]^ Systematic reviews and meta-analysis have been increasingly used to formulate public health policies and to guide resource allocation to improve population health outcomes.

The case fatality rate of MH has decreased to less than 5% with dantrolene therapy and advanced intraoperative monitoring techniques.^[7]^ However, the costs involved with continuous temperature monitoring and stocking dantrolene owing to its 3-year shelf-life limit, as well as the relatively high cost of the drug can also be issues that may govern the formulation of public health policies.^[8,9]^ Further, the paucity of epidemiologic data on MH leads to uncertainty regarding its true incidence, which limits analysis of cost-effectiveness.^[9]^

Thus, the primary objective of this systematic review and meta-analysis is to determine the incidence of MH in patients undergoing general anesthesia. Further, this review will attempt to evaluate trends in the incidence of MH. Data on the incidence of MH across different geographical regions, different age groups, gender, and race will also be analyzed.

## 2 Methods and analysis

Our systematic review and meta-analysis protocol was developed according to the preferred reporting items for systematic review and meta-analysis protocols (PRISMA-P) statement.^[10]^ The protocol for this review has been registered in the PROSPERO network (registration number: CRD42017076628). This systematic review and meta-analysis of the incidence of MH in patients undergoing general anesthesia will be performed according to the Meta-analysis of Observational Studies in Epidemiology (MOOSE) guidelines^[11]^ and will be reported according to the Preferred Reporting Items for Systematic reviews and Meta-Analysis (PRISMA) guidelines.^[12]^

### 2.1 Ethical issues

There is no funding agency for this study. Also this systematic review does not require ethical approval or informed consent because there will be no direct contact with individual patients, and only previously published data will be included in the review.

### 2.2 Inclusion and exclusion criteria

We propose to include studies that report the incidence of MH or provide prevalence data or the number of subjects with and without MH, from which the incidence can be calculated. Peer-reviewed, prospective cohort studies, retrospective cohort studies, cross-sectional studies, or reports issued by government organizations will be eligible for inclusion. Reference lists of included studies will be examined for additional relevant articles. No language or date restrictions will be applied. Non-peer-reviewed articles, review articles, case reports, case series, case–control studies, letters to the editor, commentaries, proceedings, laboratory science studies, and other nonrelevant studies will be excluded from analysis.

The population of interest will be patients undergoing general anesthesia across all countries. If the studies report the incidence or prevalence of MH in patients undergoing surgery or any type of anesthesia without specifying that the patients underwent general anesthesia, or if the studies report these cases in hospitalized inpatients as opposed to patients undergoing general anesthesia, we will attempt to contact the authors to ascertain the incidence of MH in patients undergoing general anesthesia. If this is unsuccessful, we will regard these studies as reports with an unspecified type of anesthesia, and will perform both pooled analysis and sensitivity analysis excluding the data from those studies. We will only include studies that report the incidence of MH and cases of MH; reports of neuroleptic malignant syndrome or hyperpyrexia of unclear etiology will be excluded.

### 2.3 Data sources

We propose to search MEDLINE, EMBASE, and Google scholar using search terms related to the incidence of MH. Search terms to be used for MEDLINE and EMBASE are presented in the appendix. Two authors will screen titles and abstracts of the retrieved articles. Reference lists will be imported to Endnote software 8.1 (Thompson Reuters, CA) and duplicate articles will be removed. Additional relevant articles will be identified by scanning reference lists of articles obtained from the original search.

### 2.4 Study selection

The titles and abstracts identified through the search strategy described above will be reviewed independently by 2 authors. To minimize data duplication due to multiple reporting, papers from the same author, organization, or country will be compared. For articles determined to be eligible based on the title or abstract, the full paper will be retrieved. Potentially relevant studies chosen by at least 1 author will be retrieved and the full text evaluated. Articles meeting the inclusion criteria will be assessed separately by 2 authors, and any disagreement will be resolved through discussion. In cases where agreement cannot be reached, the dispute will be resolved with the help of a third investigator. If authors are similar or incidence data are extracted from the same database, the study period will be assessed. If the study period overlaps, only the latest study will be included. A flow diagram for the search and selection process will be developed using the PRISMA guidelines.

### 2.5 Data extraction

Using a standardized extraction form, the following data will be extracted independently by 2 authors: study name (along with the name of the first author and year of publication), country where the study was conducted, source from which patients or study participants were selected, study design, outcome definition, anesthetic used, age, gender, race, incidence or prevalence with 95% confidence intervals (CIs) or the number of patients with MH and the total number of subjects. If information is inadequate, we will attempt to contact study authors and request for additional information. If unsuccessful, missing information will be calculated from the available data if possible. In case only prevalence is reported, we will consider prevalence as incidence because MH has a short duration and a high mortality; we will perform sensitivity analysis by excluding data from those studies. The reference list will be divided into 2 halves. Two authors will complete data extraction, 1 for each half of the reference list. Data extraction forms will be cross-checked to verify accuracy and consistency of the extracted data.

### 2.6 Study quality assessment

The quality of the studies will be independently assessed by 2 authors using the Newcastle–Ottawa scale (NOS) and the modified risk of bias tool (mROB).

NOS is a validated quality assessment instrument for nonrandomized trials that assesses 3 parameters of study quality: selection, comparability, and exposure assessment.^[13]^ The NOS assigns a maximum score of 4 for selection, 2 for comparability, and 3 for exposure, for a maximum total score of 9. Studies with a total NOS score of 5 or greater are considered to be of moderate to high quality, whereas those with an NOS score of less than 5 are considered low-quality studies.

The mROB tool will be developed and adapted from the risk of bias tool for prevalence studies developed by Hoy et al.^[14]^ This tool assigns a maximum score of 4 for external validity and 6 for internal validity (Table 1). Each item will be assigned a score of 1 (Yes) or 0 (No), and scores will be summed across items to generate an overall quality score. The total score will range from 0 to 10. Studies with an mROB score of 5 or greater are considered to be of moderate to high quality, whereas those with an mROB score of < 5 are considered low-quality studies. Any discrepancies will be resolved through discussion. If an agreement cannot be reached, the dispute will be resolved with the help of a third investigator.

**Table 1:**
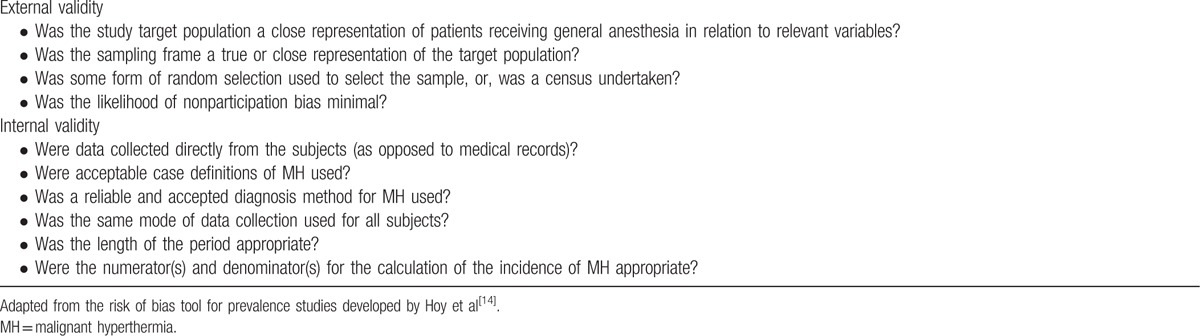
Modified risk of bias item.

### 2.7 Statistical analysis

Ad-hoc tables will be designed to summarize data from the included studies and show their key characteristics and important questions related to the aim of this review. After data extraction, reviewers will determine whether a meta-analysis is possible.

### 2.8 Data synthesis

We will calculate the incidence of MH in each study. Following this, the pooled incidence for all studies with a corresponding 95% CI will be computed.

Heterogeneity between studies will be assessed using the Cochran's *Q* and Higgins *I*^2^ statistic. A *P*-value of < .10 for the Chi^2^ statistic or an *I*^2^ > 50% will be considered as showing considerable heterogeneity, and data will be analyzed using the Mantel–Haenszel random-effect model. Under other circumstances, we will apply the Mantel–Haenszel fixed-effect model.^[15]^

### 2.9 Subgroup analysis

If data are sufficient, we will conduct subgroup analysis based on the quality of study, region, age, gender, race, and the provoking agent.

### 2.10 Sensitivity analysis

We plan to conduct sensitivity analyses to evaluate the influence of individual studies on the overall effect estimate by excluding one study at a time from the analysis. We will also perform sensitivity analysis by excluding studies reporting the incidence of MH on patients undergoing surgery with an unspecified anesthetic technique, any type of anesthesia, or in hospitalized inpatients without providing further details instead of patients undergoing general anesthesia, or those studies reporting only prevalence.

### 2.11 Publication bias

Publication bias will be assessed by using Begg's funnel plot and Egger's test. Begg's funnel plots are scatter plots of the log odds ratios (ORs) of individual studies on the *x*-axis against 1/standard error (SE) of each study on the *y*-axis. Egger's test is a test for linear regression of the normalized effect estimate (log OR/SE) against its precision (1/SE).^[16]^ An asymmetrical funnel plot or a *P*-value of < .10 on Egger's test will be considered to indicate the presence of publication bias. If publication bias is detected, trim and fill analysis will be performed. All statistical analyses will be performed using Stata SE version 15.0 (StataCorp, College Station, TX).

### 2.12 Evidence synthesis

The evidence grade will be determined using the guidelines of the GRADE (Grading of Recommendations, Assessment, Development, and Evaluation) system which uses sequential assessment of the evidence quality that is followed by an assessment of the risk–benefit balance and a subsequent judgment on the strength of the recommendations.^[17]^

## 3 Discussion

This protocol presents the methodology of a systematic review for assessing the incidence of MH in patients undergoing general anesthesia. In addition, this review will reveal the influence of geographical region, age, gender, race, type of anesthetic, and provoking agent on the incidence of MH.

To our knowledge, this review will be the first to analyze literature on the general incidence of MH. Although the most common initial signs of MH, such as hypercarbia, sinus tachycardia, masseter spasm, and elevated body temperature,^[18]^ become evident soon after anesthetic administration, we may not recognize these as early signs of MH in the absence of musculoskeletal disorders, family history of MH, and known genetic abnormalities. The main reason for conducting this systemic review and meta-analysis is to provide better insight into this fatal but clinically important complication. Overall, we believe that our systematic review will provide new information on the incidence of MH in patients undergoing general anesthesia.

### 3.1 Publication plan

This systematic review will be published in a peer-reviewed journal and will be disseminated electronically and in print.

## 4 Authors’ contributions

JI, EJA, DKL, and HK conceived this study. JI and EJA developed the study protocol and will implement the systematic review under the supervision of DKL and HK. DKL and HK will provide the statistical analysis plan of the study and will conduct data analysis. JI and EJA will perform the study search, screening, and extraction of data whereas DKL and HK will review the work. JI wrote the first manuscript draft and all authors gave input to the final draft of the protocol.

## Supplementary Material

Supplemental Digital Content
