# Supplementary material for: Incidence of malignant hyperthermia in patients undergoing general anesthesia: Protocol for a systematic review and meta-analysis
Source: Medicine (Baltimore). 2017 Dec 8;96(49):e9115. doi: 10.1097/MD.0000000000009115 (PMC5728960; doi:10.1097/MD.0000000000009115)
Supplement: Supplemental Digital Content [file medi-96-e9115-s001.docx]

**Search terms to be used**

**MEDLINE**

1. Exp malignant hyperthermia/
2. Malignant.mp.
3. Exp Anesthesia/
4. Anaesthesia.mp.
5. Anesthetic.mp.
6. Anaesthetic.mp.
7. Or/2-6
8. Hyperthermia.mp.
9. Hyperpyrexia.mp.
10. Or/8-9
11. 7 and 10
12. 1 or 11
13. Exp incidence/
14. Exp prevalence/
15. Occurrence*.mp.
16. exp epidemiology/
17. or/13-16
18. 12 and 17

**EMBASE**

1. 'malignant hyperthermia'/exp
2. Malignant
3. 'anesthesia'/exp
4. #2 OR #3
5. 'hyperthermia'/exp
6. Hyperpyrexia
7. #5 OR #6
8. #4 AND #7
9. 'incidence'/exp
10. 'prevalence'/exp
11. Occurrence
12. 'epidemiology'/exp
13. #9 OR #10 OR #11 OR #12
14. #8 AND #13
